# Association of low-dosage systemic corticosteroid use with disease burden in asthma

**DOI:** 10.1038/s41533-020-00192-x

**Published:** 2020-08-04

**Authors:** Kazuto Matsunaga, Mitsuru Adachi, Hiroyuki Nagase, Tomoko Okoba, Nobuya Hayashi, Yuji Tohda

**Affiliations:** 1 Department of Respiratory Medicine and Infectious Disease, Graduate School of Medicine, Yamaguchi University, 1-1-1 Minami-kogushi, Yamaguchi, 755-8505 Japan; 2 Clinical Research Center, International University of Health and Welfare/Sanno Hospital, 8-10-16 Akasaka, Minato-ku, Tokyo, 107-0052 Japan; 3 Division of Respiratory Medicine and Allergology, Department of Medicine, Teikyo University School of Medicine, 2-11-1 Kaga, Itabashi-ku, Tokyo, 173-8606 Japan; 4 AstraZeneca K.K., Grand Front Osaka Tower B, 3-1 Ofuka-cho, Kita-ku, Osaka, 530-0011 Japan; 5 Department of Respiratory Medicine and Allergology, Kindai University Faculty of Medicine, 377-2 Ohnohigashi, Osakasayama, Osaka, 589-8511 Japan

**Keywords:** Asthma, Asthma, Adverse effects

## Abstract

There is an ongoing debate about the benefit–risk balance of systemic corticosteroids (SCS) in asthma treatment. We investigated the associations between SCS use and disease burden in a database cohort of asthmatics, categorized into SCS and non-SCS prescription at baseline and quartiles (Q) by cumulative SCS dosage. Of the 10,579 patients, the SCS cohort comprised 3103 patients (29.3%). Mean SCS dosages at baseline were 0.08, 0.29, 0.79, and 4.58 mg/day in Q1, Q2, Q3, and Q4, respectively. Similar SCS dosages were used within each quartile throughout the study period. No remarkable changes in asthma severity or control status were observed. All SCS cohorts had a higher risk of intermittent SCS exposure during the observation period. SCS use was associated with osteoporosis, diabetes, anxiety/neurosis, and depression. SCS-dependent treatment does not necessarily lead to the future improvement of asthma control; rather, it may negatively impact systemic health, even at mean dosages <5 mg/day.

## Introduction

The World Health Organization estimated that about 235 million individuals currently have asthma worldwide^1^. The Ministry of Health, Labour and Welfare estimated that 1,177,000 patients were receiving asthma treatment in Japan in October 2014^2^. The KEIFU study, a large-scale database study, estimated that 2.5% of the adult Japanese asthma population had severe, uncontrolled asthma, and 7.8% had severe asthma^3^.

Asthma management goals are to achieve good symptom control; maintain normal activity levels; and minimize the risk of exacerbations, fixed airflow limitations, and potential treatment side effects^4^. Current guidelines^4^ accept systemic corticosteroids (SCS), that is, corticosteroids administered orally or by intramuscular injection, for the treatment of uncontrolled asthma because of their potent anti-inflammatory and immunomodulatory properties^5^. However, there is debate about the benefit–risk balance of SCS in asthma treatment. Based on current recommendations, low-dosage SCS (<7.5 mg/day) should be used to minimize the substantial and well-recognized side effects associated with SCS treatment^4^. In fact, many studies have confirmed a daily or cumulative dose–response relationship between SCS use (including long-term and repeated short-term SCS use) and the risk of steroid-related comorbidities^6–9^. It is suspected that SCS-dependent treatment does not improve asthma control. Rather, SCS may result in the development of several comorbidities and are associated with high financial costs; thus SCS may have a negative impact on patients’ health.

**Fig. 1:**
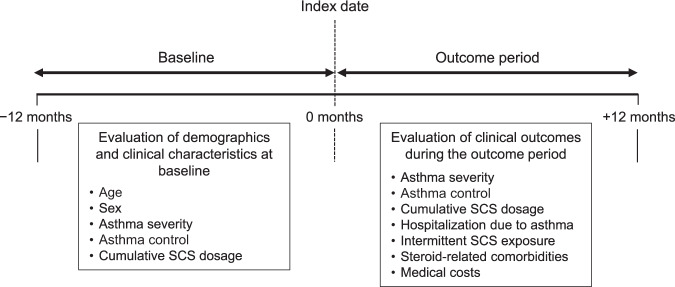
Study design. *SCS* systemic corticosteroid.

We conducted a cohort analysis of data from the non-interventional KEIFU study, which included detailed longitudinal data of health insurance claims of continuously treated Japanese asthma patients^3^. Our objectives were to evaluate the degree of exposure to SCS at baseline (12 months pre-index date) and to explore the associations between SCS use and the disease burden in asthma, including aspects such as asthma control (exacerbations), comorbidities, and medical costs, during the outcome period (12 months post-index date) (Fig. 1).

## Results

### Patient disposition

**Fig. 2:**
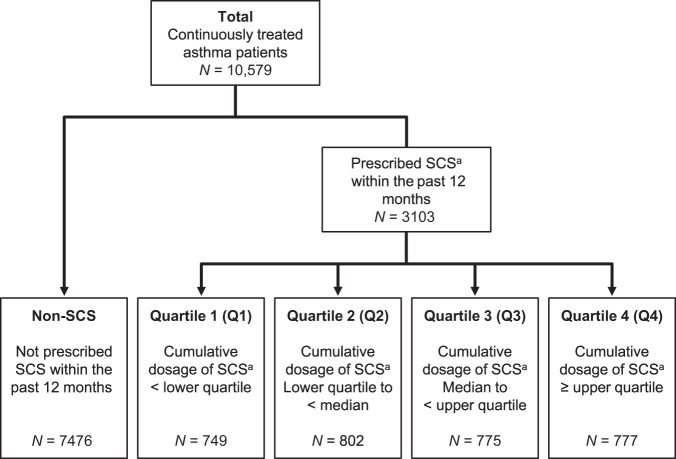
Patient disposition. ^a^Prednisone equivalent. *SCS* systemic corticosteroid.

There were 2,911,085 policyholders during the study enrolment period (1 April 2014 to 31 March 2015), of which 96,687 patients were diagnosed with asthma and prescribed inhaled corticosteroids (ICS) or ICS/long-acting beta-agonists (LABA). Of these, 10,579 patients were identified as continuously treated asthma patients and were included in this analysis. In total, 3103 continuously treated asthma patients (29.3%) were prescribed SCS at baseline. These patients were further classified into quartiles according to the cumulative dosage of SCS, which was defined as the SCS dosage prescribed for 12 months prior to the index date (Fig. 2).

### Demographics and clinical characteristics

Table 1 shows the baseline patient demographics and clinical characteristics. Overall, patients had a mean age of 48.0 years, and approximately half were women (50.1%). The percentages of patients with severe asthma (4.4%, 8.7%, 11.7%, and 45.7%); severe, uncontrolled asthma (1.5%, 1.6%, 4.3%, and 22.8%); and severe controlled asthma (2.9%, 7.1%, 7.5%, and 22.9%) increased from quartile 1 (Q1) to Q4, respectively. Mean SCS dosages and mean total number of days on which SCS were administered were 0.08, 0.29, 0.79, and 4.58 mg/day and 3.96, 8.27, 24.86, and 133.60 days in Q1, Q2, Q3, and Q4, respectively.

**Table 1:** Patient demographics and clinical characteristics at baseline.

	All patients	Non-SCS	Q1	Q2	Q3	Q4
	*N* = 10,579	*N* = 7476	*N* = 749	*N* = 802	*N* = 775	*N* = 777
Age						
Mean (SD), years	48.0 (12.2)	48.0 (12.3)	46.7 (12.0)	47.6 (11.9)	47.8 (12.1)	50.3 (12.0)
Sex						
Male, *n* (%)	5277 (49.9)	3955 (52.9)	328 (43.8)	330 (41.1)	316 (40.8)	348 (44.8)
Female, *n* (%)	5302 (50.1)	3521 (47.1)	421 (56.2)	472 (58.9)	459 (59.2)	429 (55.2)
Asthma severity and control status						
Severe asthma	823 (7.8)	274 (3.7)	33 (4.4)	70 (8.7)	91 (11.7)	355 (45.7)
Uncontrolled, *n* (%)	267 (2.5)	33 (0.4)	11 (1.5)	13 (1.6)	33 (4.3)	177 (22.8)
Controlled, *n* (%)	556 (5.3)	241 (3.2)	22 (2.9)	57 (7.1)	58 (7.5)	178 (22.9)
Mild-to-moderate asthma	9756 (92.2)	7202 (96.3)	716 (95.6)	732 (91.3)	684 (88.3)	422 (54.3)
Uncontrolled, *n* (%)	1646 (15.6)	637 (8.5)	210 (28.0)	224 (27.9)	293 (37.8)	282 (36.3)
Controlled, *n* (%)	8110 (76.7)	6565 (87.8)	506 (67.6)	508 (63.3)	391 (50.5)	140 (18.0)
Daily SCS dosage during 12 months before the index date						
Mean (SD), mg/day	0.42 (1.69)	0 (0)	0.08 (0.04)	0.29 (0.08)	0.79 (0.25)	4.58 (4.42)
Total number of days on which SCS were administered during 12 months before the index date^a^						
Mean (SD), days	12.54 (52.71)	0 (0)	3.96 (4.97)	8.27 (11.90)	24.86 (47.13)	133.60 (138.05)

*SD* standard deviation, *SCS* systemic corticosteroid, *Q* quartile.

^a^Excluding intermittent use of SCS.

### Association between SCS use and disease burden in asthma

The mean prescribed dosages of SCS for Q1–Q4 are shown in Supplementary Fig. 1. Notably, the mean dosages prescribed before and after the index date were similar in each of the subgroups from Q1 to Q4.

The severity and control status of asthma before and after the index date remained the same for most of the study population (Supplementary Table 1). Asthma severity was consistent in 78.0% of patients with severe asthma and 94.7% of patients with mild-to-moderate asthma. In 93.3% of patients, asthma severity remained the same throughout the study period. Of the patients with severe, controlled asthma; mild-to-moderate controlled asthma; severe, uncontrolled asthma; and mild-to-moderate uncontrolled asthma before the index date, 68.6%, 90.4%, 61.1%, and 65.1% of patients, respectively, had the same asthma severity and control status after the index date.

**Fig. 3:**
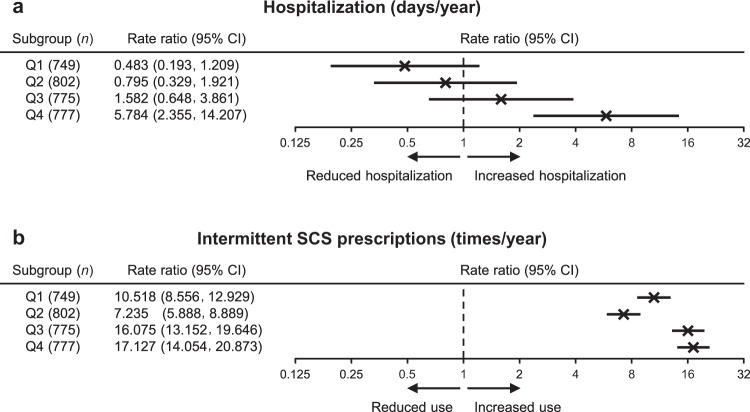
Adjusted rate ratios of asthma exacerbation-related events according to cumulative systemic corticosteroid dosage. Quartiles shown for **a** hospitalization due to asthma and **b** intermittent SCS prescriptions. *CI* confidence interval, *Q* quartile, *SCS* systemic corticosteroid.

Figure 3 shows SCS use and its impact on asthma exacerbation-related events. Even after adjusting for confounders, there was an association between SCS use and hospitalization in Q3 and Q4, with a more marked association in Q4 (adjusted rate ratio = 5.78, 95% confidence interval [CI]: 2.36, 14.21) (Fig. 3a). Moreover, there was a strong association between intermittent SCS use, defined as the intermittent prescription of SCS in the prescription record, and cumulative SCS dosage in all the four subgroups (Fig. 3b and Supplementary Table 2). The mean number of intermittent SCS prescriptions was 1.04 times in Q1, 0.73 times in Q2, 1.60 times in Q3, and 1.77 times in Q4 (Supplementary Table 2). The number of intermittent exposures to SCS was markedly increased in all subgroups compared with the non-SCS cohort (individually adjusted rate ratios = 7.24–17.13).

### Associations between SCS, comorbidities, and medical costs

**Fig. 4:**
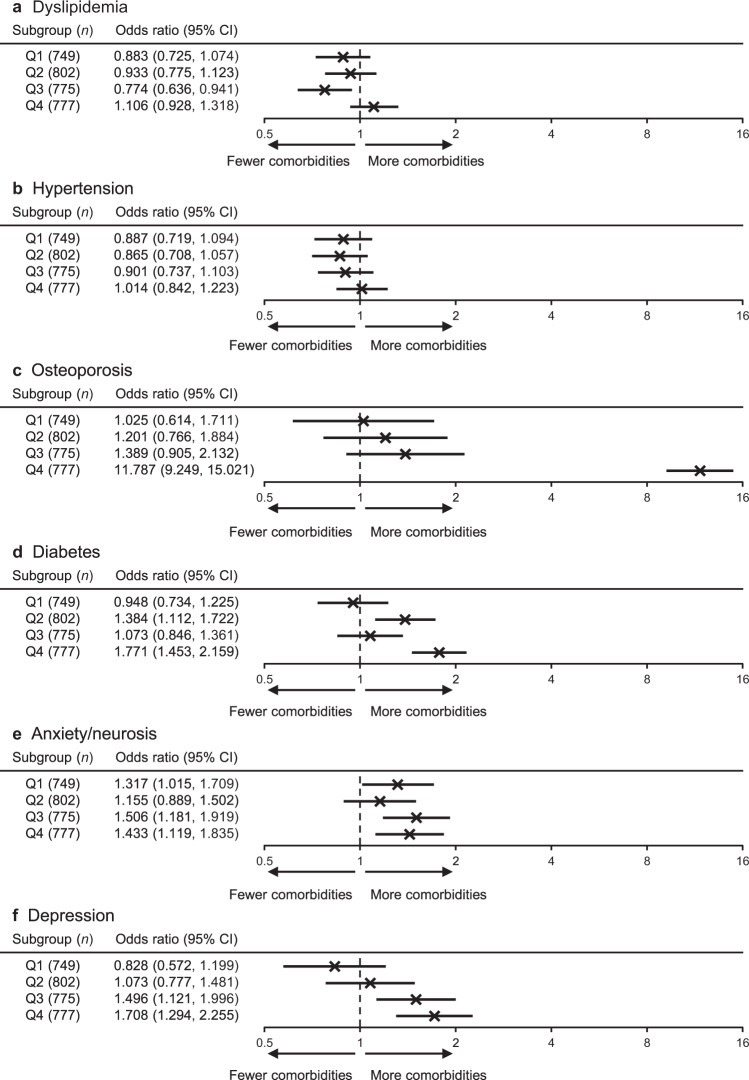
Adjusted odds ratios of corticosteroids-related comorbidities according to cumulative systemic corticosteroid dosage. Quartiles shown for **a** dyslipidemia, **b** hypertension, **c** osteoporosis, **d** diabetes, **e** anxiety/neurosis, and **f** depression. *CI* confidence interval, *Q* quartile.

**Fig. 5:**
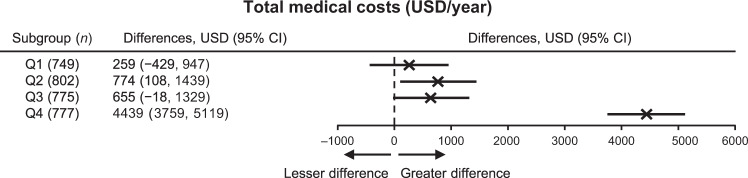
Difference in total medical costs according to the quartiles by cumulative systemic corticosteroid dosage. *CI* confidence interval, *Q* quartile, *USD* United States dollars.

According to the logistic regression model (Fig. 4), osteoporosis and anxiety/neurosis were associated with SCS use in patients of all subgroups. However, the association between osteoporosis and SCS use for patients in Q4 was very strong (adjusted odds ratio [OR] = 11.79; 95% CI: 9.25, 15.02) compared with that in other subgroups. Diabetes and depression were associated with SCS use in Q2, Q3, and Q4. Supplementary Table 3 shows the percentages of comorbidities by quartile. Total medical costs were highest in Q4 compared with the other subgroups, with an adjusted difference of 4439 United States dollars (USD)/year (95% CI: 3759, 5119 USD) vs. non-SCS patients (Fig. 5 and Supplementary Table 4). Regarding drug treatment costs for asthma and comorbidities (Supplementary Table 5), costs were the highest for drugs to treat asthma.

## Discussion

We evaluated the associations between SCS use and disease burden in asthma; specifically, asthma control (exacerbations), steroid-related comorbidities, and medical costs. SCS were prescribed as asthma treatment to approximately 30% of patients. In this study, we analyzed both continuous use and intermittent use of SCS by quartile, and the average dosage of SCS was <5 mg/day, meeting the “low dose” criteria of the Global Initiative for Asthma (GINA) guidelines^4^. In each subgroup, SCS dosage was similar before and after the index date; nevertheless, there were no remarkable changes in the severity and control status of asthma throughout the study period. In patients with SCS exposure, associations were observed between low-dosage SCS use and risks of exacerbation-related events, comorbidities, and higher medical costs, demonstrating a negative impact of SCS exposure. This is consistent with the results of the previous studies from the standpoint of the burden of both continuous use and intermittent use^6,7,9–12^.

Exacerbation-related events, including hospitalization and intermittent SCS prescriptions, occurred in many patients who had used SCS in the previous year compared with those who had not. This finding is consistent with the findings of a previous report in which prior SCS use was the strongest predictor of future SCS use and was associated with greater asthma burden, including asthma exacerbation and uncontrolled asthma^13,14^. Although short-term SCS treatment is needed as rescue treatment for asthma exacerbation, sustained associations were observed between low-dosage SCS use and risk of exacerbation-related events. When compared by SCS dosage, no major difference was observed in the number of intermittent uses of SCS. Considering SCS use, there was no change between the dosage prescribed in the previous year and that prescribed 12 months later, suggesting that these patients were treated with similar SCS dosages. SCS use may not lead to future suppression or prevention of asthma exacerbation, regardless of dosage and duration.

In patients with mild-to-moderate asthma, the percentages of uncontrolled vs. controlled asthma were 28.0% vs. 67.6% in Q1, 27.9% vs. 63.3% in Q2, and 37.8% vs. 50.5% in Q3, showing predominance of well-controlled patients, but 36.3% vs. 18.0% in Q4. Thus the control status in Q1–Q3 differed from that in Q4. The severity and control status of asthma between the baseline period and the outcome period remained the same for most study patients. Over 60% of patients who had severe uncontrolled asthma during the baseline period had severe uncontrolled asthma during the outcome period. This suggests that similar SCS-dependent treatment may not improve severity or majorly affect asthma control. In a previous study of Japanese adult patients with severe asthma who were followed up for 10 years, a significant relationship was observed between the progressive loss of lung function and oral corticosteroid use^15^. An evidence-based evaluation of six published studies concluded that, in patients with asthma exacerbations, SCS use did not improve airflow limitation or reduce the need for hospitalization^16^. Asthma is recognized as a heterogeneous disease with different phenotypes^17^. The inefficacy of the higher SCS doses may be attributable to the presence of steroid-resistant phenotypes^18^. Therefore, alternative treatments, other than SCS, to manage severe, uncontrolled asthma and to decrease or eliminate SCS exposure are required^19^. Recently, the steroid-sparing effect of biologics such as omalizumab, mepolizumab, benralizumab, and dupilumab has been studied^20^. GINA guidelines currently recommend the use of some biologics for patients with severe asthma (Step 5)^4^.

Although this study cannot demonstrate causality, relevant associations were identified between SCS use, including intermittent use and continuous use, and steroid-related comorbidities in continuously treated Japanese asthma patients. These results are relevant as we analyzed SCS use at mean dosages <5 mg/day. Regarding intermittent SCS use, our outcomes are similar to those of a previous study, which showed that intermittent oral corticosteroid use greater than four times was related to an increase in corticosteroid adverse events (AEs)^8^. Regarding continuous SCS use, the risk of AEs during 12 months of treatment with 7.5 mg/day SCS use is widely accepted as low^4^. Many studies have shown that steroid-induced AEs are dose and duration dependent^21–23^; however, the present study demonstrated that, even with a mean dosage <5 mg/day, SCS use was associated with increased corticosteroid-related comorbidities and total medical costs. Our outcomes are consistent with a recent review that concluded that the reduction of SCS dosage may be insufficient to ameliorate AE burden, which in turn increases medical costs in asthma patients with long-term exposure to SCS^5^.

Total medical costs were the highest among patients in Q4 (mean difference of 4439 USD). This was attributable to costs related to hospitalization, outpatient care, management of comorbidities, and asthma treatment. Our findings are consistent with those of a recent study using data from a medical claims database, in which the increased likelihood of comorbidities related to SCS use translated into elevated annual medical costs estimated to be 2712–8560 USD above those of non-users^12^. Similarly, a real-life asthma study in Sweden reported that the total yearly health care resource utilization cost for SCS users was threefold than that of non-SCS users^24^.

This study has limitations. Given the nature of the data assessed, only associations between SCS use and each outcome were evaluated, so causality was not established. Our results might not be an accurate depiction of the severity, control status, or adherence status of patients as the data used do not include information on patients’ symptoms, the results of laboratory tests, or adherence checks. The database does not include data on elderly individuals (aged ≥75 years). A previous study^25^ observed that comorbidities associated with SCS treatment were not evenly distributed across age or sex, which may have influenced the study results. In addition, we analyzed SCS use for 1 year at baseline and throughout the outcome period; therefore, our study did not evaluate the short-term effect of SCS for asthma control. Despite these weaknesses, our study exposes potential adverse effects of SCS-dependent treatment, including low-dose SCS.

In conclusion, our study showed that, in patients with asthma, SCS-dependent treatment does not necessarily lead to future improvement of asthma control; rather, it may have a negative impact on patients’ health, even when the mean administered SCS doses were low. Further studies are required to verify whether alternative treatments not depending on SCS can attenuate the burdens of asthma on systemic health and medical costs.

## Methods

### Study patients

Detailed eligibility criteria have been published^3^. Patients who met all of the following criteria were defined as having continuously treated asthma and were eligible for analysis: age ≥17 years at the index date; had at least one record of claims data within 12 months before the index date; had a diagnosis of asthma (confirmed if the medical record listed the International Classification of Diseases-10 codes J45 or J46) at ≥12 months before the index date; and had at least four visits for asthma with prescription of ICS or an ICS/LABA between 1 April 2014 and 31 March 2015, as summarized in Fig. 1. The index date was the date of the latest visit for asthma at which an ICS or ICS/LABA was prescribed within the abovementioned period. Patients not prescribed asthma treatment with ICS or ICS/LABA within 6 months or between 7 and 12 months before the index date or during a ≥6-month interval between any two visits for asthma were excluded.

### Study design

This was an observational cohort analysis of data from the KEIFU study. The KEIFU study design has previously been published^3^. Data for this study were retrieved in July 2017 from a health insurance claims database, updated and managed by JMDC Inc. (Tokyo, Japan), containing longitudinal, anonymized data of health insurance claims (inpatient, outpatient, and pharmacy) and check-ups for all insured persons (i.e., employees and their insurance-covered family members aged <75 years) from >90 health insurance unions (~3.7 million people or 2.5% of Japan’s total population). The study protocol and its amendments were approved by the Takahashi Clinic Institutional Review Board. The need for informed consent was waived by the Takahashi Clinic Institutional Review Board because this was a non-interventional study, and patient data obtained from the claims database were anonymized. The study was conducted in accordance with Ethical Guidelines for Biomedical Research Involving Human Subjects, the ethical principles of the Declaration of Helsinki, and all relevant regulations applicable to non-interventional studies. This study was registered in the University Hospital Medical Information Network (UMIN): UMIN000027695.

### Definition of subgroups by cumulative SCS dosage

Patients were categorized by SCS prescription at baseline. The prescription status of SCS was classified into quartile subgroups according to the cumulative dosage of SCS at which the SCS was prescribed for 12 months prior to the index date (Q1, minimum [0–25th percentile] cumulative dosage, to Q4, maximum [75th–100th percentile] dosage). In addition, both continuous use and intermittent use were analyzed for Q1, Q2, Q3, and Q4.

### Evaluation of asthma severity and control status

Asthma severity and control status were evaluated at baseline and during the outcome period. Patients with severe asthma were defined as patients prescribed a 1600-μg/day budesonide-equivalent dosage or a greater ICS dosage plus at least one controller (LABA, leukotriene modifier, or theophylline) or having ≥183 prescription days for SCS within 12 months (Supplementary Table 6). Patients were defined as having uncontrolled asthma if they met at least one of the following criteria during 12 months: (1) prescription of short-acting β_2_-agonists for >208 attacks; (2) ≥two prescriptions of short-term use oral steroids and/or injectable steroids; and/or (3) hospitalization for asthma with at least one injectable steroid prescription. Definitions were based on the European Respiratory Society/American Thoracic Society guidelines^26^. To compare the paired data before and after the index date, the analysis of asthma control and severity was conducted only in cases where these multifaceted decision criteria were definitively verified.

### Associations between SCS, exacerbations, and medical costs

Associations between SCS use and exacerbation-related events (hospitalization due to asthma and intermittent use of SCS), total medical costs, and drug treatment costs related to asthma and comorbidities were assessed during the outcome period. In this study, intermittent use, which was defined as intermittent prescription of SCS in the prescription records, was considered equivalent to “as needed” use. Medical costs for up to 12 months starting from the month after the index date were tabulated. Costs (yen/month) were calculated and converted to USD (1 yen = 0.0087846917 USD [27 October 2017]).

### Evaluation of the association of SCS use with comorbidities

Data on diseases, including hypertension, dyslipidemia, osteoporosis, diabetes, anxiety/neurosis, and depression, were used. SCS prescription status data at the index date were recorded, and the number of corresponding prescriptions was calculated by multiplying the number of prescriptions per month in the main analysis by 12 months. Associations between SCS use and percentage of comorbidities were evaluated.

### Statistical analysis

Claims data without exact prescription or assessment dates were replaced with those for the first day of the prescription month or assessment month. Other missing data were not replaced. Statistical analyses were conducted using IBM Netezza Analytics (IBM Netezza, Marlborough, MA, USA) and SAS version 9.3 (SAS Institute Inc., Cary, NC, USA). Outcomes and demographic/clinical characteristic data were summarized using descriptive statistics.

Associations between SCS use and comorbidities were evaluated using a logistic regression model, and the adjusted ORs and their 95% CIs were presented. The impact of SCS use on exacerbation-related events was evaluated based on a negative binomial regression model, and the adjusted rate ratios and their 95% CIs were presented. The impact of SCS use on medical costs was evaluated based on a linear model, and the adjusted differences and their 95% CIs were presented. All models included SCS prescription status, age, and sex as covariates. Unadjusted results are also presented.

### Reporting summary

Further information on experimental design is available in the Nature Research Reporting Summary linked to this article.

## Supplementary information

Reporting Summary

Supplementary Information

## Data Availability

Data underlying the findings described in this manuscript may be obtained in accordance with AstraZeneca’s data sharing policy described at http://astrazenecagrouptrials.pharmacm.com/ST/Submission/Disclosure.
