# Supplementary material for: Association of low-dosage systemic corticosteroid use with disease burden in asthma
Source: NPJ Prim Care Respir Med. 2020 Aug 4;30:35. doi: 10.1038/s41533-020-00192-x (PMC7403577; doi:10.1038/s41533-020-00192-x)
Supplement: Supplementary file 1 — Reporting Summary [file 41533_2020_192_MOESM1_ESM.pdf]

nature research

Corresponding author(s): Kazuto Matsunaga, MD, PhD

Last updated by author(s): Jun 12, 2020

Reporting Summary

Nature Research wishes to improve the reproducibility of the work that we publish. This form provides structure for consistency and transparency in reporting. For further information on Nature Research policies, see Authors & Referees and the Editorial Policy Checklist .

Statistics

For all statistical analyses, confirm that the following items are present in the figure legend, table legend, main text, or Methods section.

n/a	Confirmed	
☐	☒	The exact sample size (*n*) for each experimental group/condition, given as a discrete number and unit of measurement
☒	☐	A statement on whether measurements were taken from distinct samples or whether the same sample was measured repeatedly
☐	☒	The statistical test(s) used AND whether they are one- or two-sided<br>*Only common tests should be described solely by name; describe more complex techniques in the Methods section.*
☐	☒	A description of all covariates tested
☐	☒	A description of any assumptions or corrections, such as tests of normality and adjustment for multiple comparisons
☐	☒	A full description of the statistical parameters including central tendency (e.g. means) or other basic estimates (e.g. regression coefficient) AND variation (e.g. standard deviation) or associated estimates of uncertainty (e.g. confidence intervals)
☒	☐	For null hypothesis testing, the test statistic (e.g. *F*, *t*, *r*) with confidence intervals, effect sizes, degrees of freedom and *P* value noted<br>*Give P values as exact values whenever suitable.*
☒	☐	For Bayesian analysis, information on the choice of priors and Markov chain Monte Carlo settings
☒	☐	For hierarchical and complex designs, identification of the appropriate level for tests and full reporting of outcomes
☒	☐	Estimates of effect sizes (e.g. Cohen's *d*, Pearson's *r*), indicating how they were calculated

Our web collection on statistics for biologists contains articles on many of the points above.

Software and code

Policy information about availability of computer code

Data collection	Data for this study were retrieved in July 2017 from a health insurance claims database, updated and managed by JMDC Inc. (Tokyo, Japan).
Data analysis	IBM Netezza Analytics N2002-010 7.1.0.4.P2 and SAS version 9.3 (SAS Institute Inc., Cary, North Carolina, USA)

For manuscripts utilizing custom algorithms or software that are central to the research but not yet described in published literature, software must be made available to editors/reviewers. We strongly encourage code deposition in a community repository (e.g. GitHub). See the Nature Research guidelines for submitting code & software for further information.

Data

Policy information about availability of data

All manuscripts must include a data availability statement. This statement should provide the following information, where applicable:
- Accession codes, unique identifiers, or web links for publicly available datasets
- A list of figures that have associated raw data
- A description of any restrictions on data availability

Data underlying the findings described in this manuscript may be obtained in accordance with AstraZeneca’s data sharing policy described at http://astrazenecagrouptrials.pharmacm.com/ST/Submission/Disclosure

Field-specific reporting

Please select the one below that is the best fit for your research. If you are not sure, read the appropriate sections before making your selection.

Life sciences study design

All studies must disclose on these points even when the disclosure is negative.

Sample size	Data for this study contained longitudinal, anonymized data of health insurance claims (inpatient, outpatient, and pharmacy) and check-ups for all insured persons (i.e., employees and their insurance-covered family members aged ≤74 years) from over 90 health insurance unions (~3.7 million people or 2.5% of Japan's total population).
Data exclusions	Patients not prescribed asthma treatment with inhaled corticosteroid (ICS) or ICS/long-acting beta-agonists (LABA) within 6 months or between 7 and 12 months before the index date, or during a ≥6-month interval between any two visits for asthma were excluded.The index date was the date of the latest visit for asthma at which an ICS or ICS/LABA was prescribed between 1 April 2014 and 31 March 2015.
Replication	n/a
Randomization	This was an observational cohort analysis of data from the previously published KEIFU study. Data for this study were retrieved from a health insurance claims database.
Blinding	This was an observational cohort analysis of data from the previously published KEIFU study. Data for this study were retrieved from a health insurance claims database.

Reporting for specific materials, systems and methods

We require information from authors about some types of materials, experimental systems and methods used in many studies. Here, indicate whether each material, system or method listed is relevant to your study. If you are not sure if a list item applies to your research, read the appropriate section before selecting a response.

Materials & experimental systems

n/a	Involved in the study	
☒	☐	Antibodies
☒	☐	Eukaryotic cell lines
☒	☐	Palaeontology
☒	☐	Animals and other organisms
☐	☒	Human research participants
☐	☒	Clinical data

Methods

n/a	Involved in the study	
☒	☐	ChIP-seq
☒	☐	Flow cytometry
☒	☐	MRI-based neuroimaging

Human research participants

Policy information about studies involving human research participants

Population characteristics	Male or female aged ≥17 years at the index date; had at least one record of claims data within 12 months before the index date; had a diagnosis of asthma (confirmed if the medical record listed the ICD-10 codes J45 or J46) at ≥12 months before the index date; and had at least four visits for asthma with prescription of inhaled corticosteroids (ICS) or an ICS/long-acting beta-agonists (LABA) between 1 April 2014 and 31 March 2015. The index date was the date of the latest visit for asthma at which an ICS or ICS/LABA was prescribed within the abovementioned period.
Recruitment	Data for this study were retrieved from a health insurance claims database.
Ethics oversight	The study protocol and its amendments were approved by the Takahashi Clinic Institutional Review Board. The study was conducted in accordance with Ethical Guidelines for Biomedical Research Involving Human Subjects, the ethical principles of the Declaration of Helsinki, and all relevant regulations applicable to non-interventional studies. The need for informed consent was waived by the Takahashi Clinic Institutional Review Board because this was a non-interventional study, and patient data obtained from the claims database were anonymized .

Note that full information on the approval of the study protocol must also be provided in the manuscript.

Clinical data

Policy information about clinical studies

All manuscripts should comply with the ICMJEguidelines for publication of clinical research and a completedCONSORT checklist must be included with all submissions.

Clinical trial registration	University Hospital Medical Information Network (UMIN): UMIN000027695
Study protocol	Study protocol in English is not available. Because study protocol was written in Japanese language. If there is request, protocol information in English will be shared.

Data collection	Data for this study were retrieved from a health insurance claims database.
Outcomes	Following patient proportion in treated asthma patients;severe asthma, uncontrolled severe asthma
