# Supplementary material for: Association of low-dosage systemic corticosteroid use with disease burden in asthma
Source: NPJ Prim Care Respir Med. 2020 Aug 4;30:35. doi: 10.1038/s41533-020-00192-x (PMC7403577; doi:10.1038/s41533-020-00192-x)
Supplement: Supplementary file 2 — Supplementary Information [file 41533_2020_192_MOESM2_ESM.pdf]

Supplementary Figure 1. Mean prescribed SCS dosage before and after the index date

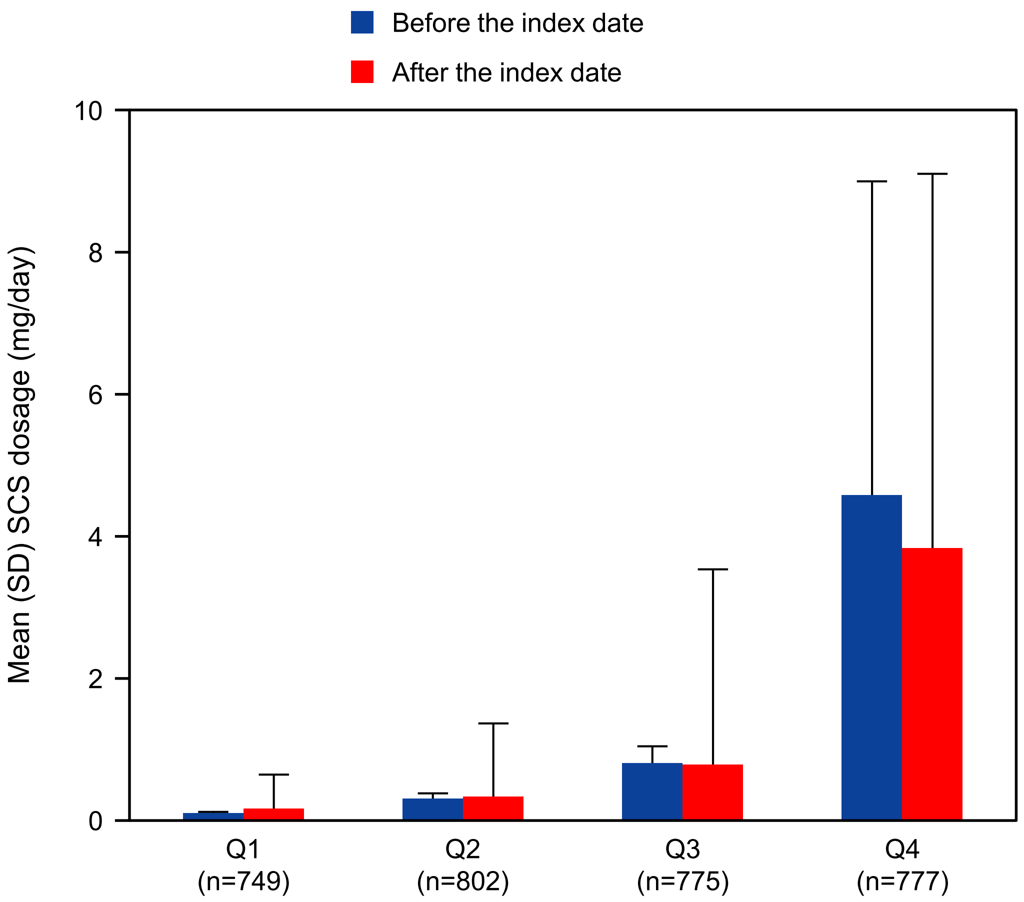

Error bars in the figure represent SD.
Q quartile, *SCS* systemic corticosteroids, *SD* standard deviation

Supplementary Table 1. Asthma severity and control status before and after the index date

		Severity and control status after the index date					
		Severe asthma			Mild to moderate asthma		
		Total	Uncontrolled	Controlled	Total	Uncontrolled	Controlled
Severity and control status before the index date		*n* (%)	*n* (%)	*n* (%)	*n* (%)	*n* (%)	*n* (%)
Severe asthma	*N* = 581[a]	453 (78.0)	151 (26.0)	302 (52.0)	128 (22.0)	28 (4.8)	100 (17.2)
Uncontrolled	*N* = 193[a]	154 (79.8)	118 (61.1)	36 (18.7)	39 (20.2)	23 (11.9)	16 (8.3)
Controlled	*N* = 388[a]	299 (77.1)	33 (8.5)	266 (68.6)	89 (22.9)	5 (1.3)	84 (21.6)
Mild to moderate asthma	*N* = 6,190[a]	326 (5.3)	76 (1.2)	250 (4.0)	5,864 (94.7)	976 (15.8)	4,888 (79.0)
Uncontrolled	*N* = 1,118[a]	88 (7.9)	55 (4.9)	33 (3.0)	1,030 (92.1)	728 (65.1)	302 (27.0)
Controlled	*N* = 5,072[a]	238 (4.7)	21 (0.4)	217 (4.3)	4,834 (95.3)	248 (4.9)	4,586 (90.4)

[a]The analysis of asthma control and severity was conducted only in cases where the multifaceted decision criteria according to the definitions in the European Respiratory Society/American Thoracic Society guidelines were definitively verified.

Supplementary Table 2. Exacerbation-related events

	Non-SCS<br>*N* = 7,476	Q1<br>*N* = 749	Q2<br>*N* = 802	Q3<br>*N* = 775	Q4<br>*N* = 777
Hospitalization					
Mean (SD), days/year	0.78 (11.41)	0.37 (3.08)	0.63 (4.16)	1.21 (15.16)	4.58 (25.89)
Unadjusted rate ratio (95% CI)	Reference	0.475<br>(0.191–1.181)	0.802<br>(0.333–1.930)	1.545<br>(0.636–3.749)	5.844<br>(2.392–14.273)
Adjusted rate ratio (95% CI)	Reference	0.483<br>(0.193–1.209)	0.795<br>(0.329–1.921)	1.582<br>(0.648–3.861)	5.784<br>(2.355–14.207)
Intermittent SCS prescriptions					
Mean (SD), times/year	0.10 (0.55)	1.04 (4.04)	0.73 (2.98)	1.60 (13.30)	1.77 (3.25)
Unadjusted rate ratio (95% CI)	Reference	10.308<br>(8.389–12.665)	7.300<br>(5.946–8.961)	15.923<br>(13.051–19.427)	17.632<br>(14.466–21.492)
Adjusted rate ratio (95% CI)	Reference	10.518<br>(8.556–12.929)	7.235<br>(5.888–8.889)	16.075<br>(13.152–19.646)	17.127<br>(14.054–20.873)

CI confidence interval, *SCS* systemic corticosteroids, *SD* standard deviation, *Q* quartile

Supplementary Table 3. Corticosteroid-related comorbidities

	Non-SCS<br>*N* = 7,476	Q1<br>*N* = 749	Q2<br>*N* = 802	Q3<br>*N* = 775	Q4<br>*N* = 777
Dyslipidaemia					
n (%)	1,778 (23.8)	152 (20.3)	176 (21.9)	151 (19.5)	219 (28.2)
Unadjusted odds ratio (95% CI)	Reference	0.816 (0.678–0.983)	0.901 (0.756–1.074)	0.776 (0.644–0.934)	1.258 (1.066–1.484)
Adjusted odds ratio (95% CI)	Reference	0.883 (0.725–1.074)	0.933 (0.775–1.123)	0.774 (0.636–0.941)	1.106 (0.928–1.318)
Hypertension					
n (%)	1,653 (22.1)	138 (18.4)	152 (19.0)	154 (19.9)	195 (25.1)
Unadjusted odds ratio (95% CI)	Reference	0.796 (0.656–0.965)	0.824 (0.685–0.991)	0.874 (0.726–1.051)	1.180 (0.995–1.401)
Adjusted odds ratio (95% CI)	Reference	0.887 (0.719–1.094)	0.865 (0.708–1.057)	0.901 (0.737–1.103)	1.014 (0.842–1.223)
Osteoporosis					
n (%)	169 (2.3)	17 (2.3)	23 (2.9)	26 (3.4)	169 (21.8)
Unadjusted odds ratio (95% CI)	Reference	1.004 (0.606–1.663)	1.277 (0.821–1.986)	1.501 (0.986–2.284)	12.018 (9.561–15.106)
Adjusted odds ratio (95% CI)	Reference	1.025 (0.614–1.711)	1.201 (0.766-1.884)	1.389 (0.905–2.132)	11.787 (9.249–15.021)
Diabetes					
n (%)	852 (11.4)	75 (10.0)	114 (14.2)	91 (11.7)	154 (19.8)
Unadjusted odds ratio (95% CI)	Reference	0.865 (0.674–1.110)	1.288 (1.044–1.590)	1.034 (0.822–1.302)	1.922 (1.589–2.325)
Adjusted odds ratio (95% CI)	Reference	0.948 (0.734–1.225)	1.384 (1.112–1.722)	1.073 (0.846–1.361)	1.771 (1.453–2.159)
Anxiety/Neurosis					
n (%)	533 (7.1)	72 (9.6)	70 (8.7)	86 (11.1)	82 (10.6)
Unadjusted odds ratio (95% CI)	Reference	1.385 (1.070–1.794)	1.246 (0.960–1.616)	1.626 (1.278–2.069)	1.537 (1.203–1.964)
Adjusted odds ratio (95% CI)	Reference	1.317 (1.015–1.709)	1.155 (0.889–1.502)	1.506 (1.181–1.919)	1.433 (1.119–1.835)
Depression					
n (%)	368 (4.9)	32 (4.3)	44 (5.5)	58 (7.5)	64 (8.2)
Unadjusted odds ratio (95% CI)	Reference	0.862 (0.596–1.247)	1.121 (0.813–1.546)	1.562 (1.172–2.083)	1.734 (1.315–2.286)
Adjusted odds ratio (95% CI)	Reference	0.828 (0.572–1.199)	1.073 (0.777–1.481)	1.496 (1.121–1.996)	1.708 (1.294–2.255)

CI confidence interval, *SCS* systemic corticosteroids, *Q* quartile

Supplementary Table 4. Total medical costs

	Non-SCS<br>*N* = 7,476	Q1<br>*N* = 749	Q2<br>*N* = 802	Q3<br>*N* = 775	Q4<br>*N* = 777
Total medical costs					
Mean, USD/year (95% CI)	3,224<br>(3,017–3,431)	3,403<br>(2,744–4,061)	3,968<br>(3,333–4,603)	3,871<br>(3,228–4,514)	7,847<br>(7,197–8,497)
Unadjusted difference,<br>USD/year (95% CI)	Reference	179<br>(−512–869)	744<br>(77–1,412)	647<br>(−29–1,323)	4,623<br>(3,941–5,305)
Adjusted difference, USD/year (95% CI)	Reference	259<br>(−429–947)	774<br>(108–1,439)	655<br>(−18–1,329)	4,439<br>(3,759–5,119)

CI confidence interval, *SCS* systemic corticosteroids, *Q* quartile, *USD* United States dollars

Supplementary Table 5. Drug treatment costs of asthma and comorbidities

	Non-SCS<br>*N* = 7,476	Q1<br>*N* = 749	Q2<br>*N* = 802	Q3<br>*N* = 775	Q4<br>*N* = 777
Asthma drug					
Mean, USD/year (95% CI)	571<br>(554–588)	618<br>(564–672)	737<br>(685–789)	837<br>(783–890)	1,244<br>(1,190–1,297)
Unadjusted difference, USD/year (95% CI)	Reference	48<br>(−9–104)	166<br>(111–221)	266<br>(210–322)	673<br>(617–729)
Adjusted difference, USD/year (95% CI)	Reference	50<br>(−7–107)	166<br>(111–221)	264<br>(208–320)	661<br>(605–717)
Asthma drug (except SCS)					
Mean, USD/year (95% CI)	570<br>(553–587)	615<br>(561–669)	733<br>(681–785)	827<br>(774–880)	1,203<br>(1,150–1,256)
Unadjusted difference, USD/year (95% CI)	Reference	46<br>(−11–102)	164<br>(109–218)	257<br>(202–313)	633<br>(577–689)
Adjusted difference, USD/year (95% CI)	Reference	48<br>(−9–105)	163<br>(108–218)	255<br>(200–311)	622<br>(565–678)
Hypertension drug					
Mean, USD/year (95% CI)	61<br>(57–65)	51<br>(37–64)	54<br>(41–67)	59<br>(46–72)	66<br>(53–79)
Unadjusted difference, USD/year (95% CI)	Reference	−10<br>(−24–3)	−7<br>(−20–6)	−2<br>(−15–12)	5<br>(−9–19)
Adjusted difference, USD/year (95% CI)	Reference	−4<br>(−17–9)	−3<br>(−16–10)	2<br>(−11–15)	−1<br>(−14–12)
Dyslipidaemia drug					
Mean, USD/year (95% CI)	38<br>(35–40)	34<br>(25–43)	31<br>(23–40)	28<br>(20–37)	55<br>(46–64)
Unadjusted difference, USD/year (95% CI)	Reference	−4<br>(−13–5)	−6<br>(−16–3)	−9<br>(−18–0)	17<br>(8–27)
Adjusted difference, USD/year (95% CI)	Reference	−1<br>(−11–8)	−5<br>(−14–4)	−9<br>(−18–0)	13<br>(3–22)
Diabetes drug					
Mean, USD/year (95% CI)	36<br>(32–41)	34<br>(19–48)	35<br>(21–50)	29<br>(15–44)	42<br>(27–56)
Unadjusted difference, USD/year (95% CI)	Reference	−2<br>(−18–13)	−1<br>(−16–14)	−7<br>(−22–8)	6<br>(−10–21)
Adjusted difference, USD/year (95% CI)	Reference	1<br>(−15–16)	1<br>(−13–16)	−5<br>(−20–10)	2<br>(−13–18)

CI confidence interval, *SCS* systemic corticosteroids, *Q* quartile, *USD* United States dollars

Supplementary Table 6. Definition of asthma severity and control status

Category	ERS/ATS criteria	Items used for criteria of control in this study (by using data available in the database)	
		Use of ERS/ATS criteria without alteration	Conversion from ERS/ATS criteria
Severe asthma	GINA[4] Step 4–5	×	Prescribed high-dosage ICS plus at least one other controller
	Use of systemic corticosteroids ≥50%	Number of days of prescriptions for systemic corticosteroids ≥183	–
Severe, uncontrolled asthma	ACQ >1.5<br>ACT <20	×	Number of SABA prescription records (≥208 attacks)[a]
	Short-term use of systemic corticosteroids ≥2 times (>3 times each)	×	At least two prescription records for short-term use of systemic corticosteroids or injectable corticosteroids[b]
	Hospitalization admission to ICU mechanical ventilation ≥1 time	Number of hospitalizations for asthma[c] (emergency visits cannot be used)	–
	Airflow limitation	×	×

ERS/ATS European Respiratory Society and American Thoracic Society, *ICU* intensive care unit, *ICS* inhaled corticosteroids, *LABA* long-acting β_2-agonist, *SABA* short-acting β_2-agonist, *OCS* oral corticosteroids, *GINA* Global Initiative for Asthma, *ACQ* Asthma Control Questionnaire, *ACT* Asthma control test

[a]Refers to a description of one criterion of level of asthma symptom control in GINA 2017 – “Reliever needed more than twice/week (in the past 4 weeks)” – and considering real-world usage of SABA as 50% of each formulation. The number of inhalations per attack depends on the formulation.

[b]Refers to patients with at least two prescription records for short-term use of systemic corticosteroids or injectable corticosteroids ≤12 months prior to the index date. However, a minimum 14-day interval between the relevant record and the previous record was required.

[c]With at least one prescription of injectable corticosteroids.

×: data in the database could not be used as originally recorded. In these cases, criteria were altered so that data compiled in the database could be used (shown in the right column).

–: conversion of criteria was not necessary because data from the database could be used as originally recorded.

Note that the criterion for airflow limitation could not be appropriately converted and therefore was not used in this study.
